# Measuring emotions triggered by fragrance through behavioral paradigms

**DOI:** 10.3389/fpsyg.2025.1468416

**Published:** 2025-06-20

**Authors:** Yue Lv, Hua Sun, Hong-yu Wu, Kaiwen Xiong

**Affiliations:** Shanghai China-norm Quality Technical Service Co., Ltd., Shanghai, China

**Keywords:** fragrance, emotion, behavioral paradigms, EEG, SAM

## Abstract

**Intoduction:**

Fragrance has a positive impact on various aspects of people’s emotions, experiences, and behaviors, such as product satisfaction, happiness, and comfort in daily life. While previous research has predominantly utilized physiological measurements or subjective evaluations, behavioral methods have been underexplored. This study aims to explore the measurement of emotional responses to fragrance through behavioral paradigms.

**Methods:**

In a self-controlled experiment, participants were instructed to smell various fragrances and assessed their emotional changes before and after exposure using EEG, emotion scales, and behavioral paradigms.

**Results:**

The findings revealed that the emotional bias measured by the Go/No-go Association Task (GNAT) was consistent with the emotional valence results obtained from EEG measurements, and the trend of time perception measured by the time bisection task was consistent with the arousal index measured by EEG. After smelling the orange fragrance, the EEG valence of participants increased from 2.61 to 3.68 (*p* = 0.017), the d value increased from −0.66 to 0.21 (*p* = 0.071), the EEG arousal decreased from 0.97 to −0.30 (*p* = 0.092), and the temporal dilation value increased from 57.24 to 67.51 (*p* = 0.038).

**Discussion:**

The results indicate that changes in emotional valence and arousal in response to fragrance can be effectively assessed through behavioral paradigms. These results contribute to the fragrance product development and provide insights for enhancing multisensory experiences.

## 1 Introduction

Previous studies have shown that fragrance profoundly impacts emotional states, which can positively affect a person’s sense of happiness and satisfaction in their daily living environment (Torriani et al., 2024; Xiao et al., 2021; Spence, 2020). In recent years, the fragrance industry has increasingly focused on improving emotions through fragrance. In related scientific studies, researchers have commonly utilized a combination of physiological measurements and self-assessment to investigate the impact of fragrance on emotions. Xiong et al. (2023) employed electroencephalogram (EEG) and mood scales to demonstrate that exposure to the natural aroma of jasmine led to a significant decrease in β waves associated with excitement or alertness in participants, indicating a reduction in tension and stress. Park et al. (2019) also observed notable differences in EEG patterns following exposure to different scents. Their findings revealed that inhaling lavender scent resulted in emotionally stabilizing effects, characterized by an increase in α waves (associated with relaxation), a decrease in β waves (linked to excitement and alertness), and an increase in α/β ratios (indicative of arousal levels). Conversely, exposure to mint fragrance produced contrasting results, with a decrease in α waves, an increase in β waves, and a decrease in α/β ratio, indicating an arousing effect. Springer et al. (2022) explored the impact of scented skincare products on emotions and reported similar outcomes, specifically an increase in α wave activity signifying a relaxation effect. Recently, a growing body of EEG research employed machine learning or deep learning methods to investigate classification of emotional state based on frequency domain features, with reported accuracy surpassing 80% for validation and testing (Adhikari et al., 2025; Fernandes et al., 2024). Furthermore, researchers have found that citrus and sweet orange essential oils can help reduce emotional stress after mental exertion, as evidenced by measurements of blood pressure, heart rate, and heart rate variability (Liu et al., 2023; Wang et al., 2023). Notably, Painchault et al. (2020) integrated behavioral measurements as non-verbal assessment tools alongside physiological measurements in their investigation of the relaxation response to scented shampoo. They identified differences in reaction time between the treatment and control groups, underscoring the importance of behavioral measurement tools in assessing emotional responses. However, limited studies have employed behavioral tools to assess emotional responses.

Behavioral paradigms, also known as behavioral tasks or experimental models, serve as tools to assess various aspects such as cognition, emotions, or attitudes in a standardized manner. Behavioral paradigms offer a convenient non-verbal means of measurement distinct from physiological and subjective scales (Nosek and Banaji, 2001). These paradigms contribute to enhancing the existing dimensions of emotional measurement, thereby enriching the indicators related to emotions. Drawing from Russell’s two-dimensional emotional model (Russell, 1980), emotions are characterized by independent variations in valence (pleasure-displeasure) and arousal (degree of arousal). In the realm of behavioral research, some studies have explored the connection between emotions and temporal perception. For instance, Baccarani et al. employed a time bisection task and observed that participants tended to underestimate time duration when exposed to relaxed and soothing odors while overestimating it in the presence of stimulating odors. Subsequent research further validated the efficacy of the time bisection task in elucidating the relationship between time perception and arousal mechanisms (Baccarani et al., 2021; Baccarani and Brochard, 2023). Moreover, certain studies have utilized implicit association tests to gauge the association between a target object and positive or negative emotions, thereby revealing individuals’ emotional inclinations toward specific concepts. Ge et al. (2019) employed the Go/No-go Association Task (GNAT), a variant of the implicit paradigm, to investigate the processing of emotional information among groups with varying levels of social adaptation. This paradigm, integrating the principles of signal detection theory, assesses participants’ ability to recognize signals through d-scores (Nosek and Banaji, 2001). The findings indicated that groups with higher levels of social adaptation exhibited greater recognition of positive emotional information compared to negative emotional information, suggesting a preference for positive emotions. Conversely, groups with lower levels of social adaptation demonstrated the opposite pattern.

The present study employed electroencephalography (EEG) and self-assessment as comparative methodologies. In EEG analysis of emotional responses, the disparity in α waves between the left and right frontal lobes is commonly associated with motivational tendencies (approach and withdrawal). Numerous studies utilize the difference in α waves between the left and right frontal lobes to signify emotional valence, indicating the extent of positive or negative emotions, and utilize the β/α ratio to compute the arousal index (Gabriel et al., 2021). In self-assessment techniques, evaluating emotional valence and arousal dimensions is a prevalent approach; however, it is imperative to elucidate the indicator concepts to the participants. Some researchers have devised image-based scales to mitigate language comprehension discrepancies, such as the SAM Emotion Scale, Affective Slider, EmoCards, and other instruments, which have undergone extensive utilization and validation (Ping et al., 2024).

In summary, combining behavioral paradigms to measure odor-related emotional responses is a promising area of exploration, although existing research is limited. The objective of this research is to assess the emotional reactions elicited by fragrances using a behavioral paradigm. Previous research has indicated that citrus essential oils (e.g., wild orange (sweet orange), bergamot, grapefruit, etc.) have a positive impact on boosting positive emotions, whereas mint has an arousing effect (Agarwal et al., 2022; Park et al., 2019). Based on these findings, this study employed sweet orange and mint essential oils as olfactory stimuli to evaluate changes in emotional valence and arousal.

Chapter 2 provides a detailed introduction to the research methodology, including participant recruitment and screening, fragrance stimulation methods, measurement methods, and procedure; Chapter 3 elaborates on the measurement results; Chapter 4 discusses the meaning and limitations of existing results based on previous research; Chapter 5 summarizes the research.

## 2 Materials and methods

### 2.1 Participants

A total of 35 participants within the age range of 23–55 were enrolled in the study, of whom 33 completed the experiment, including 21 females and 12 males. Before their sessions, participants underwent screening to ensure the absence of allergies, and airway congestion (due to cold/flu) and were instructed to refrain from consuming alcoholic beverages, coffee, tea, cigarettes, sleeping pills, or any other substances that could impact their mental state within 24 h of the session. Additionally, they were advised to avoid using strongly scented products. In the screening process, to ensure that the participants have normal olfactory function and to exclude individuals who reject the test fragrance, the participants were asked to evaluate four different essential oil perfumes and classify them into likes, dislikes, or neutral according to their personal preferences. Two of these perfumes, orange and mint, are used for official testing. All participants were of Chinese descent and were recruited locally. Ethical approval for this study was obtained from the Shanghai Clinical Research Ethics Committee, adhering to the principles of the Helsinki Declaration. Informed consent was obtained from all participants, and they were compensated appropriately upon completion of the experiment.

### 2.2 Fragrance stimulation

The fragrance products examined in this research are commercially available products that have been officially registered with the National Medical Products Administration of China. In the experimental phase, a non-aqueous aromatherapy device was utilized to disperse the fragrance, and each participant was exposed to the scent for 8 min while resting with their eyes shut. In previous studies, the duration of continuous stimulation in environmental odor experiments ranged from 2 to 30 min (Xiong et al., 2023; Fang et al., 2025; Li et al., 2024). Considering the adaptability of the senses, we did not use nasal input, but instead used a waterless aromatherapy machine to diffuse the fragrance in the environment. The testing procedure was standardized as adding 5 drops of essential oil to the aromatherapy machine at the beginning of the day, and adding 2 drops every 4 h. During the experiment, the aromatherapy machine was placed 20 cm in front of the participants. And in the pre-testing screening stage, participants who felt repelled by the test fragrance were excluded, further avoiding the risk of sensory adaptation stress caused by the fragrance. Secondly, considering the participants’ fatigue with the experimental process and the duration of effective data, the duration of each participant’s exposure to the fragrance environment was set to 8 min.

### 2.3 EEG measurement

The Emotiv EPOC X is a wireless, portable EEG device featuring 14 channels based on the 10–20 system. It utilizes a saline electrode cap and operates at a sampling rate of 256SPS. The raw data collected from the device was imported into EEGLAB (Delorme and Makeig, 2004) 2023 in MATLAB R2023b for preprocessing and batch processing. Electrode placement followed the international 10–20 system standards, and a non-causal, zero-phase FIR bandpass filter between 0.1 and 64 Hz and a notch filter between 48 and 52 Hz were applied to remove baseline drift and power-line interference. The Spherical algorithm was implemented for interpolating any faulty channels and excluding any defective segments. Then all EEG data were segmented into non-overlapping 2-s epochs and processed via Independent Component Analysis (ICA) to identify and eliminate artifacts (Jung et al., 2000). Finally, the Fast Fourier Transform was utilized to convert the signals into the frequency domain for spectral analysis. The power spectral density of α and β waves was calculated to obtain the key metrics in this study: emotional valence [α (F4 + F8 + AF4)-α(AF3 + F3 + F7)] and arousal index [β/α (F4 + F8 + AF4 + AF3 + F3 + F7)].

### 2.4 Emotion scale

The SAM scale is utilized for assessing the subjective emotions of participants both before and after exposure to incense, specifically focusing on pleasure (valence) and arousal. To enhance participants’ comprehension, the evaluation dimensions are elucidated through picture annotations. Each dimension is rated using a Likert 9-point scale, ranging from 1 to 9. Post-incense exposure, the fragrance concentration is also evaluated using a Likert 9-point rating to determine the effectiveness of fragrance dispersion. Given that time perception and arousal measurement in this study are predicated on the same valence of fragrance, the pleasure level induced by the fragrance post-exposure is evaluated. If the concentration rating falls below 3 points, the on-site staff must verify the effectiveness of fragrance dispersion. Should the fragrance dispersion be deemed normal, the data outcomes will be utilized to determine their validity. Participants exhibiting a pleasure rating below 5 points, deviating from the overall trend in the data, will be excluded.

### 2.5 Behavioral paradigms

#### 2.5.1 Go/no-go association task

The Go/No-go Association Test (GNAT) is a variant of the implicit associative paradigm designed for implicit attitude assessment. The fundamental concept of GNAT involves prompting participants to respond to a target signal (Go stimulus) while refraining from responding to an interference signal (No-go stimulus). The difference between the hit rate (number of correct responses/total number of Go stimuli) and the false alarm rate (number of incorrect responses/total number of No-go stimuli) is transformed into a standard score known as the discriminant index (d) of the target signal. This discriminant index (d) reflects the participant’s capacity to recognize the target signal. In cases where the target signal comprises evaluation object and attribute words, a higher discriminant index (d) value indicates a stronger ability to recognize the object and attribute, signifying superior implicit recognition of the target signal by the participant. This implies a stronger association between the evaluation object and attribute.

This study consists of five stages, as delineated in Table 1. The initial stage involves the classification of emotional words, where participants are tasked with categorizing emotional words and entering responses via the keyboard without time constraints. This stage aims to familiarize participants with the vocabulary to be encountered in subsequent assessments and to assess the test’s effectiveness based on error rates. The second stage acts as a preparatory phase for the third stage, using “fragrance + positive emotion words” as the Go stimulus and “fragrance + negative emotion words” as the No-go stimulus. Participants are directed to respond to the target signal (“fragrance + positive emotion words”) while ignoring the interference signal (”fragrance + negative emotion words time limit of 1,500 ms. Upon completion, participants are informed that the time limit for formal testing will be reduced (the vocabulary utilized in this stage will not reappear in the third stage). The third stage involves measuring the discrimination ability of the same procedures as the second stage, with a time limit of 800 ms. The fourth stage serves as a rehearsal for the fifth stage, employing “fragrance + negative emotion words” as the Go stimulus and “fragrance + positive emotion words” as the No-go stimulus, with a time limit of 1,500 ms. The fifth stage is designed to assess the discriminative ability of “fragrance + negative emotion words” following the testing procedures of the fourth stage. The experimental protocol was developed and presented using E-prime 3.0, and the testing order of stages 2, 3, 4, and 5 was counterbalanced across participants.

**TABLE 1:** GNAT program settings.

Stage	Block	f	j	Space	Not responding
1	Practice	Fragrance + positive emotion words	Fragrance + negative emotion words		
2	Practice			Fragrance + positive emotion words	Fragrance + negative emotion words
3	Test			Fragrance + positive emotion words	Fragrance + negative emotion words
4	Practice			Fragrance + negative emotion words	Fragrance + positive emotion words
5	Test			Fragrance + negative emotion words	Fragrance + positive emotion words

The program utilizes vocabulary from the Geneva Emotion and Odor Scales (GEOS) and the Pleasure Scale in the Befindlichkeitsskalen (BFS) to assess participants’ pleasure levels. Positive and negative emotion words associated with pleasure (positive words—relaxed, comfor le, joyful, be well, pleasant, delighted, joyous; negative words—disgust, aversion, tension, discomfort, nausea, boredom, uncomfortable) are selected. To ensure consistency and avoid subjective interpretations that could impact reaction times, participants are instructed to provide categorical judgments on positive and negative aspects during the test.

The measurement should be conducted promptly following baseline and fragrance testing, utilizing the discriminant index d as the analytical parameter. The variance in *d*-values between the conditions of “fragrance + positive emotion words” and “fragrance + negative emotion words” was assessed to determine the level of emotional positivity exhibited by participants in various fragrance settings.

#### 2.5.2 Time bisection task

The time bisection task is used to assess participants’ time perception. In this test, two anchoring durations- one long and one short- are presented, prompting participants to distinguish whether the subsequent stimulus duration is long or short. The metrics are typically derived from projected ratios or probabilities.

During the experiment, participants were exposed to white noise through headphones and were directed to categorize and evaluate the duration of the auditory stimuli. Their reactions were documented by pressing the assigned keys (“f” for a short duration and “j” for a long duration) on the keyboard. The assignment of response keys for short and long durations was evenly distributed among all participants.

The time bisection task consists of two stages: the practice stage and the testing stage. During the practice phase, participants are required to evaluate the duration of two types of stimuli: short (300 ms) and long (900 ms). Initially, the two durations are presented randomly five times each, and participants are instructed to categorize each sound presentation as either short or long. In the testing phase, participants are presented with five different durations of sound (ranging equidistantly between 300 and 900 ms) for classification: 400, 500, 600, 700, and 800 ms, with each duration displayed ten times in a random sequence. Participants are explicitly notified that the test commences upon perceiving the fragrance.

Following the olfactory assessment, the task measurement is promptly carried out, with the temporal dilation effect (TD) serving as the analytical parameter. A non-linear function is fitted in MATLAB, with the duration of sound stimulation plotted on the x-axis and the probability of being perceived as a long-term event on the y-axis, to determine the bisection point (BP). The BP represents the point at which an event is predicted to be perceived as long-term with a 50% probability. The temporal dilation effect is then calculated as TD = 600-BP.

### 2.6 Procedure

**FIGURE 1:**
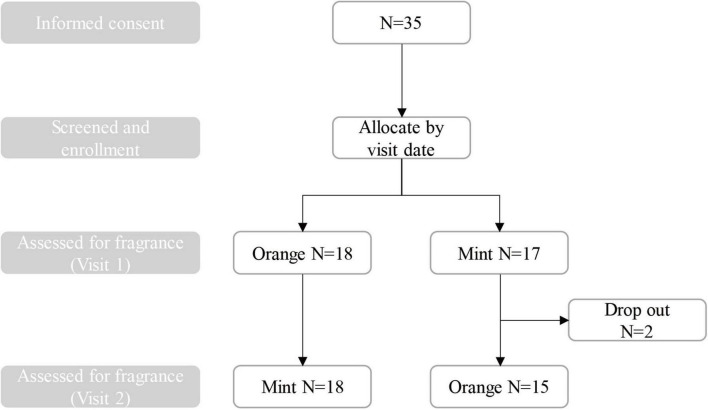
The flowchart of the study.

The experiment was conducted in the laboratory of Shanghai China-norm Quality Technical Service Co., Ltd. The indoor temperature during testing was 26°C. There are a total of 2 rooms used for experiments every day, one room without fragrance is used to collect baseline data, and the other room with fragrance is rotated by day. Each participant will undergo two visits, with each visit involving the testing of a single fragrance. Following the initial visit, participants will review the informed consent form in conjunction with the research staff. If there are no objections and they consent to participate, they will proceed to the pretest screening phase. Subsequently, participants will be formally included in the study and will engage in behavioral tasks, EEG data collection, and questionnaire completion under the supervision of the research team. They will then undergo EEG data collection while being exposed to fragrance samples, followed by a repeat of questionnaire assessments and behavioral tasks. The initial visit will last between 40 and 60 min, with a mandatory minimum interval of 24 h before the second visit. The process will be repeated for each fragrance tested, with a daily rotation of the fragrances used in the laboratory. Figure 1 shows the flow chart of participant involvement throughout the research period.

### 2.7 Statistical analysis

Statistical analysis was conducted using Statistical Product and Service Solutions (SPSS) version 22, including descriptive statistics on all data. Paired sample *t*-tests were employed for data that followed a normal distribution and EEG data, whereas Wilcoxon tests were utilized for data that did not adhere to a normal distribution.

## 3 Results

All participants achieved a minimum score of 3 points in the assessment of the concentration of sweet orange and mint fragrances, with average values of 5.48 ± 1.54 and 6.24 ± 1.23, respectively, indicating successful stimulation by the fragrances. The pleasant scores for the fragrances were 7.00 ± 1.52 and 7.21 ± 1.17, respectively, with no participants scoring below 5, and the data deviated from the general trend observed in the participant data. Table 2 presents the EEG, SAM scale, and behavioral paradigm data of all participants before and after fragrance exposure.

**TABLE 2:** Emotional measurement results.

Fragrance	Indicators		Various time stages				
			**Pre**	**Post**			
				**Starting**	**Intermediate (4 min)**	**Later (6 min)**	**The end of 8 min**
Orange	Valence	Valence (EEG)	2.61 ± 2.96	3.68 ± 2.44 (0–8 min)			
		Pleasant score (SAM)	6.06 ± 1.27	6.82 ± 1.57	7.24 ± 1.06	7.48 ± 1.23	7.39 ± 1.37
		d (GNAT)	−0.66 ± 1.37	/	/	/	0.21 ± 0.88
	Arousal	Arousal (EEG)	0.97 ± 2.94	−0.30 ± 3.37 (0–8 min)			
		Arousal score (SAM)	3.64 ± 1.43	4.30 ± 1.88	4.15 ± 1.72	3.82 ± 1.79	3.82 ± 2.05
		TD (Time bisection)	69.13 ± 75.34	/	/	/	46.70 ± 74.16
Mint	Valence	Valence (EEG)	2.34 ± 3.66	3.36 ± 3.36 (0–8 min)			
		Pleasant score (SAM)	6.06 ± 1.27	6.67 ± 1.45	6.91 ± 1.16	7.27 ± 1.44	7.33 ± 1.29
		d (GNAT)	−0.19 ± 2.11	/	/	/	0.49 ± 2.11
	Arousal	Arousal (EEG)	−2.08 ± 9.00	−0.39 ± 3.12 (0–8 min)			
		Arousal score (SAM)	3.42 ± 1.30	4.30 ± 1.94	4.30 ± 1.88	4.61 ± 2.42	4.82 ± 2.48
		TD (Time bisection)	57.24 ± 72.84	/	/	/	67.51 ± 68.33

### 3.1 Pleasant

**FIGURE 2:**
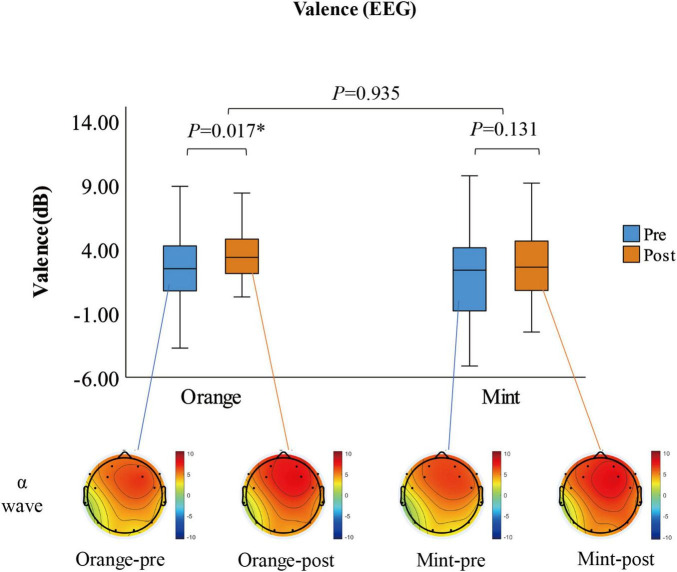
The box plot shows the Valence measured by EEG before and after smelling the fragrance, which is the hemispherical difference of α waves in the frontal lobe. The topographic map shows the distribution of α waves after the participants (*N* = 28) superimposed and averaged. *p < 0.05.

**FIGURE 3:**
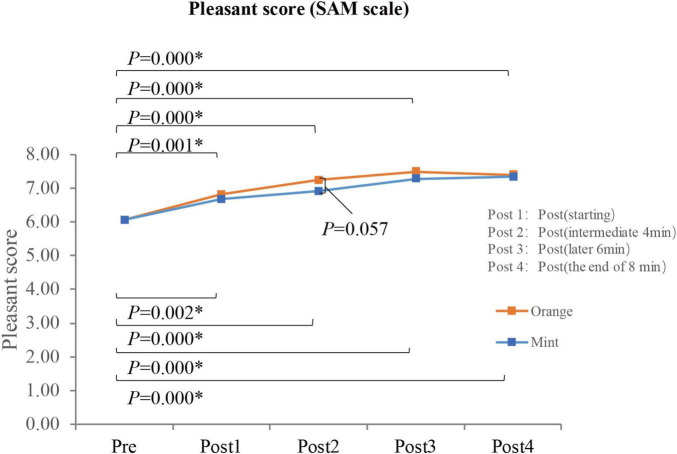
The pleasure ratings of participants (*N* = 33) at different stages before and after smelling the fragrance. *p < 0.05.

The results are presented in Figure 2. Through the utilization of a paired *t*-test, it was observed that participants experienced a noteworthy increase in Valence when exposed to the sweet orange fragrance in comparison to their baseline state (*p* = 0.017). Conversely, there was no significant alteration in Valence when exposed to the mint fragrance (*p* = 0.131). Additionally, there was no significant difference in the value of variation between the two fragrances (*p* = 0.935). Examination of the topographic map revealed a tendency for the right side of the frontal lobe α to exhibit more pronounced activity following exposure to the fragrance; however, the disparity between the effects of the two fragrances was very subtle. After 8 min of smelling the fragrance, participants were requested to retrospectively assess their feelings of joy at various intervals during the exposure. Employing the Wilcoxon test, it was determined that the pleasure ratings for both fragrances significantly increased in comparison to the baseline (*p* < 0.01), with only a notable distinction between the two fragrances emerging midway through the exposure (*p* = 0.057), as illustrated in Figure 3.

**FIGURE 4:**
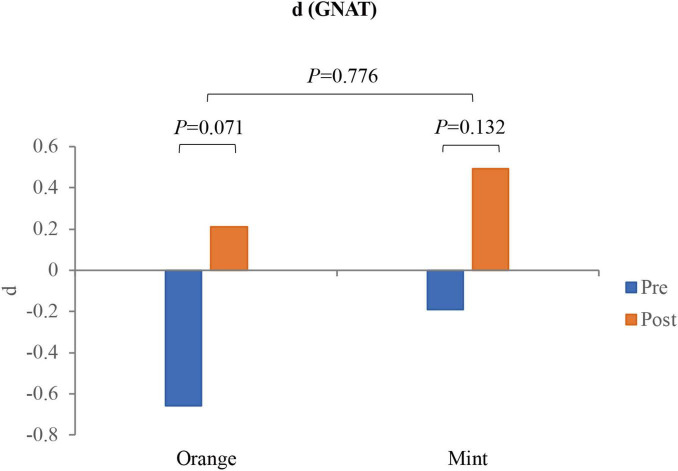
The *d*-values of participants (*N* = 17) measured using GNAT before and immediately after smelling (8 min).

The alterations in data patterns, as assessed through a behavioral paradigm, exhibit similarities to EEG patterns. Paired *t*-tests and Wilcoxon tests were conducted. Following exposure to the sweet orange fragrance, the d-value transitioned from a negative baseline to a positive value, signifying a marginal difference (*p* = 0.071), suggesting an enhancement in implicit bias toward pleasurable emotions. Conversely, exposure to the mint fragrance also demonstrated a comparable pattern; however, the observed difference was not statistically significant (*p* = 0.132). Furthermore, there was no significant distinction between the effects of the two fragrances (*p* = 0.776), as illustrated in Figure 4.

### 3.2 Arousal

**FIGURE 5:**
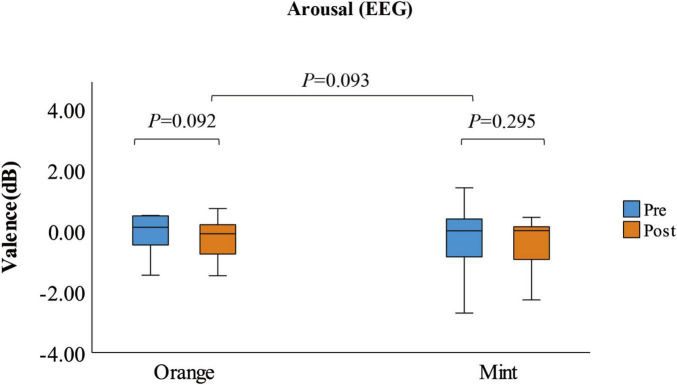
The box plot shows the arousal measured by EEG before and after smelling the fragrance (*N* = 28).

**FIGURE 6:**
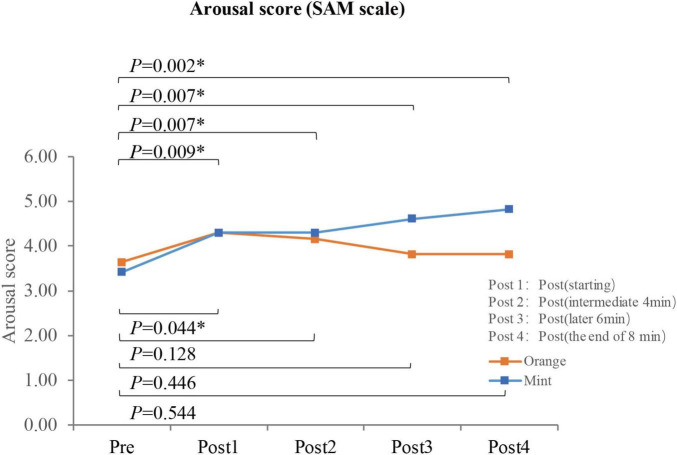
The arousal ratings of participants (*N* = 33) at different stages before and after smelling the fragrance. *p < 0.05.

Using a paired *t*-test, the arousal index of participants decreased when exposed to the sweet orange fragrance compared to the baseline, showing a critical difference (*p* = 0.092). There was no significant change in the arousal index when participants were exposed to the mint fragrance (*p* = 0.295). However, the overall distribution was more concentrated, and there was almost no significant difference between the two fragrances (*p* = 0.093), as illustrated in Figure 5. After 8 min of exposure to the fragrances, participants retrospectively assessed their arousal levels at various stages of exposure. Utilizing the Wilcoxon test, it was observed that the arousal score of participants continued to significantly increase when exposed to the mint fragrance (*p* < 0.01). In contrast, the arousal score initially increased when exposed to the sweet orange fragrance, but then decreased to the baseline level, as depicted in Figure 6. This suggests that the arousal level increases upon initial exposure to the fragrance and that the effects of different fragrances gradually manifest over time.

**FIGURE 7:**
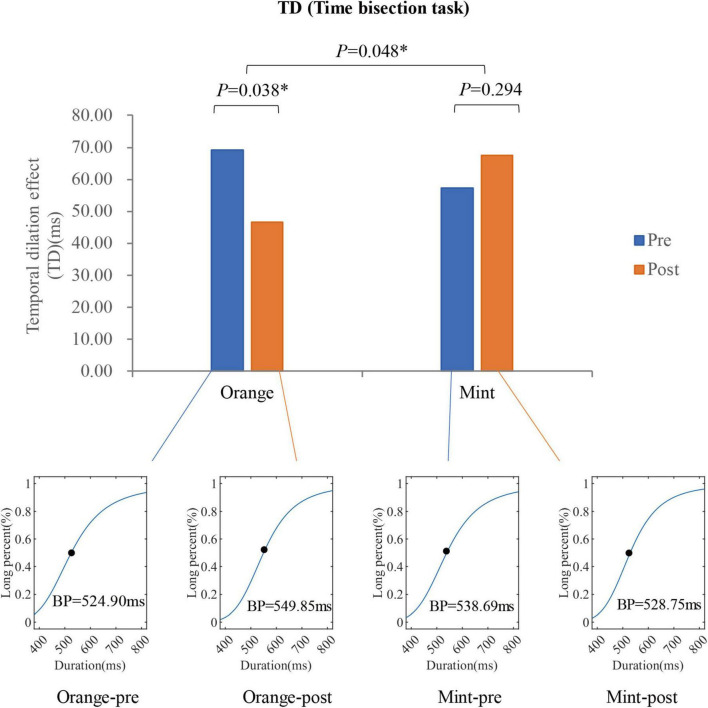
The time perception of participants (*N* = 17) measured using time tasks before and after smelling the scent (8 min). The bar graph shows the temporal dilation effect (TD), and the four line-charts show the fitting curves of time * percentage before and after smelling two different fragrances. *p < 0.05.

The Trust Region algorithm was employed to model a non-linear function using the duration of a time task as the independent variable and the probability of predicting the duration as a long duration as the dependent variable to determine the bisection point (BP), which is predicted as a long duration with a 50% probability. Before exposure to the sweet orange fragrance, the BP was 524.90 ms, which increased to 549.85 ms immediately after an 8-min exposure to the sweet orange fragrance. Preceding exposure to the mint fragrance, the BP was 538.69 ms, and following an 8-min exposure to the mint fragrance, it decreased to 528.75 ms. These findings suggest that participants tended to underestimate the objective time after exposure to the sweet orange fragrance and overestimate it after exposure to the mint fragrance. The paired *t*-test revealed a significant decrease in the TD value after exposure to the sweet orange fragrance (*p* = 0.038) and a non-significant increase after exposure to the mint fragrance (*p* = 0.294). There was a notable difference in TD changes induced by the two fragrances (*p* = 0.048). In line with the theory of time perception associated with arousal mechanisms, the results suggest a significant decrease in arousal following exposure to the sweet orange fragrance, while arousal increased without reaching statistical significance after exposure to the mint fragrance. The arousal level following exposure to the sweet orange fragrance was significantly lower compared to that following exposure to the mint fragrance. The results are depicted in Figure 7.

## 4 Discussion

### 4.1 GNAT

In the measurement of pleasure, we analyzed the emotional valence through EEG measurement, evaluated subjective pleasure through the SAM scale, and measured the bias of the measurer toward pleasure and negative emotions through GNAT. The results from the GNAT and EEG indicated an increase in the potency of the sweet orange fragrance, with no significant change in the mint fragrance. These findings suggest that assessing the pleasure effects of fragrances from a behavioral perspective is feasible to a certain extent. In a study conducted by Lemercier-Talbot et al., two fragrances with distinct emotional effects were used for implicit measurement. The study revealed that the association between scent and emotion could be quantified, such as the link between vanilla and relaxation, and between mint and energy (Lemercier-Talbot et al., 2019). While Lemercier-Talbot et al.’s research focused on measuring different arousal effects within the same valence dimension, our study contributes additional evidence that the valence distinctions of fragrances can also be assessed through implicit paradigms, a topic that has been scarcely addressed in prior research.

One aspect that warrants further investigation in the findings is the significance of validity in EEG and scale scores, contrasting with the critical significance of GNAT. Several potential reasons may account for this discrepancy. Firstly, due to operational errors or non-compliance issues, some participants may provide invalid data, resulting in a decrease in the final sample size. Although the final sample size did not fall below the initially calculated minimum (based on a two-sided paired t-test with a 95% power and a significance level (α) of 0.05, calculated using the d index of the GNAT paradigm, a mean paired difference of 0.5, and an estimated standard deviation of paired differences of 0.52, yielding a calculated sample size of 17) this reduction may have led to statistically insignificant differences in the results. Another plausible explanation could relate to the experimental design, which might have oversimplified the program trials, potentially compromising the accuracy of the measurements. Our research focused on assessing immediate emotions, which are known to be susceptible to fluctuations and external interference. To mitigate the emotional burden associated with paradigm tasks, we restricted each block of GNAT to 30 trials. In a study by Clark et al., the ST-IAT was employed to gauge emotional responses to milk packaging, with each block consisting of 84 trials (using 6 types of milk packaging as target words); however, no significant variance was observed in the outcomes (Clark et al., 2021). This suggests the necessity for further investigation to strike a balance between trial numbers and emotional load when employing implicit paradigms to assess emotions.

The scoring results of the SAM scale reveal variations compared to physiological and behavioral tasks. The results from the GNAT and EEG indicated an increase in emotional valence in response to the sweet orange fragrance, while no significant change was observed for the mint fragrance. Although there is a subtle disparity in the efficacy of the two fragrance experiences, the statistical analysis did not show a significant difference. Nevertheless, the SAM scale scores suggest that both fragrances are perceived as pleasant with no discernible variance in intensity. This underscores the more pronounced distinctions evident in the physiological and behavioral data, highlighting the advantages of objective data measurement. The discordance between subjective and objective outcomes is not uncommon in prior research, reflecting the inherent characteristics of the three measurement methods. The EEG in this study was utilized to examine the brain waves during the olfactory stimulation; the paradigm employed aimed to assess the immediate emotional response, while a scale was employed to retrospectively measure emotions at various stages of the perfume experience, which facilitated the observation of changes in emotional intensity across different stages.

### 4.2 Time bisection task

In measuring arousal, we analyzed the arousal index through EEG, evaluated subjective arousal through the SAM scale, and measured participants’ time perception through time-bisection tasks. The arousal index demonstrates a notable decrease in arousal related to the sweet orange fragrance and an increase in arousal related to the mint fragrance, although the latter is not statistically significant. A significant disparity is observed between the arousal levels induced by the sweet orange and mint fragrances. The TD measured through the time bisection task follows a similar pattern to the arousal index, albeit with a more pronounced distinction. Previous research by various scholars has established a correlation between internal clocks and changes in arousal. For instance, Baccarani et al. conducted a study that examined the impact of different aromas on arousal changes through time bisection tasks. The findings suggest that exposure to stimulating odors heightens arousal levels, leading to an overestimation of the perceived duration (Baccarani et al., 2021). Our study findings align with this notion, supporting the use of time bisection tasks to assess the arousal effects of various fragrances.

The EEG arousal index exhibited only a slight variance, possibly due to the nature of the closed-eye test, where β waves decrease (Sowndhararajan and Kim, 2016). Consequently, there was no significant alteration in β/α levels before and after exposure to the fragrance. The data indicated a more concentrated distribution of the arousal index following exposure to the mint fragrance. Participants in the experiment mostly reported feeling less drowsy after smelling the mint fragrance, although some also mentioned feeling calmer due to the cool mint scent. This suggests that the same fragrance may elicit contrasting effects among individuals. This implies that the identical fragrance could elicit contrasting responses among various individuals, leading to slight variations in the overall arousal of participants before and after exposure to the mint scent. Furthermore, the mint aroma might induce a calming effect on certain individuals instead of an arousal effect, deviating from findings reported in prior literature (Baccarani et al., 2021; Park et al., 2019; Sattayakhom et al., 2023). It is also plausible that the arousal shift induced by the fragrance may be too subtle to be captured by the β/α ratio. Combined with the measurement results of the sense of pleasure, it becomes evident that the emotional impact disparity between the two fragrances is nuanced.

The arousal score of the SAM scale continues to exhibit discrepancies from physiological and behavioral data, despite both indicating that the arousal level elicited by the sweet orange aroma is lower compared to that of the mint aroma. However, it is inconsistent that physiological and paradigmatic results show that the difference between the two fragrances lies in the significant decrease in arousal of participants after smelling the sweet orange fragrance, while scale scoring results show that the difference lies in the significant increase in arousal of the mint fragrance. The lack of a significant change in the EEG arousal index has been discussed in the preceding paragraph, with the scale scores suggesting that participants possess a heightened subjective sensitivity to high arousal states. In the realm of emotional research, some scholars have focused on attentional bias toward emotions (Clarke et al., 2020). Todd, Rebecca, et al. highlighted the presence of biased attention toward emotionally salient events (Todd et al., 2012). Through experimental investigations, Lim, SL et al. discovered that individuals are more adept at detecting significant emotions (Lim et al., 2009). Furthermore, our study also revealed that a reduction in arousal is more readily discernible through EEG readings and behavioral responses.

### 4.3 Limitations and future research

There were some factors from the current research that merit consideration and recommendation for future research.

Firstly, this study was conducted with participants exclusively from the Shanghai region of China, therefore, further experimental validation is needed to determine whether these findings can be extended to other populations.

Secondly, while the current research has preliminarily demonstrated the feasibility of measuring emotional bias and arousal changes related to fragrance using GNAT and time bisection tasks, further research is needed to explore whether other implicit paradigms or time perception measurement paradigms can also be applied.

The results of this study indicate that there are differences in GNAT (Go/No-Go Association Task), electroencephalography, and scale measurements, and GNAT (Go/No-Go Association Task) is valuable in evaluating emotional responses to odors. EEG and scales have limitations in capturing implicit emotions. Although broadly speaking, EEG is an implicit measurement, it reflects more of the activity of the central nervous system, and olfactory processing involves sensory, memory, and other related brain regions (Wang et al., 2025; Komini et al., 2023). The complexity of EEG activity has led to continuous exploration of the correspondence between its data features and popular emotional concepts.

Furthermore, given that this study focused on exploring the application of behavioral paradigms in emotion measurement, traditional and widely adopted EEG preprocessing and analysis methods were used as comparative methodology. Future work could employ state-of-the-art EEG processing techniques (Adhikari et al., 2025; Ahmed et al., 2023; Fernandes et al., 2024) and combine behavioral measures with self-reported data to further explore the impact of fragrance on emotion. Finally, in this research, the effectiveness of the retrospective measurement of aroma may be affected due to memory decline and sensory adaptation to prolonged exposure, but this was considered in this experiment to integrate centralized measurement methods.

Future research will aim to combine the characteristics of various methods to provide a more comprehensive understanding of emotions in fragrance experiences.

## 5 Conclusion

This study examined the impact of fragrance on pleasure and arousal using EEG, scales, and behavioral paradigms. The findings indicated that the GNAT paradigm and time bisection task were effective in assessing emotional biases and arousal changes associated with fragrance. After smelling the orange fragrance, the EEG valence of participants increased from 2.61 to 3.68 (*p* = 0.017), the d value increased from −0.66 to 0.21 (*p* = 0.071), the EEG arousal decreased from 0.97 to −0.30 (*p* = 0.092), and the temporal dilation value increased from 57.24 to 67.51 (*p* = 0.038). The research demonstrated the viability of evaluating emotions induced by fragrance based on task performance in behavioral paradigms. This research provides a practical method for evaluating fragrance. It can be applied to the development of fragrance products, such as perfume, skin care products, daily necessities, and other items involving olfactory experience, and also provides a reference for the evaluation of odor satisfaction, sensory experience, pleasure, and happiness in the environment.

## Data Availability

The datasets presented in this article are not readily available because the data that support the findings of this study are available from (Shanghai China-norm Quality Technical Service Co., Ltd) but restrictions apply to the availability of these data, which were used under license for the current study, and so are not publicly available. Data are however available from the authors upon reasonable request and with permission of (Shanghai China-norm Quality Technical Service Co., Ltd). Requests to access the datasets should be directed to sunnysun@china-norm.com.
